# Exposure to the Pope's Climate Change Message Activated Convinced Americans to Take Certain Activism Actions

**DOI:** 10.1002/gch2.201600019

**Published:** 2017-06-23

**Authors:** Teresa A. Myers, Connie Roser‐Renouf, Edward Maibach, Anthony Leiserowitz

**Affiliations:** ^1^ Center for Climate Change Communication George Mason University 4400, University Drive, MS 6A8 Fairfax VA 22030 USA; ^2^ Yale School of Forestry and Environmental Studies Yale University 195 Prospect Street New Haven CT 06511 USA

**Keywords:** attitude‐behavior consistency, climate change, leadership, Pope Francis

## Abstract

Many people who are concerned about the issue of climate change do not engage in the collective action behaviors that are most likely to lead to societal‐scale solutions. Such attitude‐behavior inconsistency is a well‐documented phenomenon. This study investigates whether exposure to an effectively framed message from a highly credible source can increase the consistency between attitudes and activism behaviors among people with pre‐existing strong attitudes, particularly for behaviors that are less difficult. The release of Pope Francis' climate change encyclical, *Laudato Sí*, and subsequent visit to the United States provide an opportunity to test this research question in a natural field setting. A nationally representative, within‐subject panel survey was conducted two months prior to the release of the encyclical and again four months later, after the release and papal visit, to assess the impact of the Pope's message on Americans' climate change consumer and political advocacy behaviors. Among people who are already concerned about climate change, higher exposure to the Pope's climate change message is associated with increases in attitude‐behavior consistency for less difficult activism behaviors. The findings suggest that sustained exposure to compelling climate messages from trusted sources can increase the performance of activism behaviors.

## 1 Introduction

In the summer of 2015 Pope Francis drew international attention to the issue of climate change. In his papal encyclical, *Laudato Si*,^1^ and his public appearances in the United States, he stressed the moral importance of addressing the challenge of global warming, emphasizing the human toll that climate change has and will continue to take—particularly on the world's poor—and stressing the importance of caring for our world.

The significance of transformational leadership on climate change has been recognized, although scholarly attention to the role of leadership has been relatively limited.^2^ Cues from political elites have been found to be the strongest predictor of public opinion about climate change from 2002 to 2010^3^ and others have identified religious leaders as potential transformation agents.^4^

There are multiple reasons why the Pope's climate change message may have resonated with the American public: Pope Francis is regarded favorably by a large majority of Americans;^5^ he introduced a moral frame for climate change, which experimental research has shown to be effective in fostering a felt sense of responsibility for mitigation;^6^ and his views received a great deal of media coverage—particularly during his visit to the United States.^7^

A broad swath of the public was exposed to the Pope's climate message, regardless of their prior beliefs about the reality and threat of climate change.^8^ Survey research found that public perceptions of climate change as a moral issue increased significantly between the spring, prior to the release of the encyclical, and the fall, following the Pope's U.S. visit.^9^ Exposure influenced moral issue perceptions, which in turn influenced climate change issue engagement, after controlling for pre‐existing perceptions and engagement.^8^ Both liking and trust in the Pope increased; certainty that global warming is happening and recognition that it is harmful to humans increased; the issue became more salient, as more Americans heard about it in the media and discussed it with friends and family; and issue involvement increased in terms of personal importance and amount of thought.^9^ There were, however, only small changes in support for national climate policies or individual behavior, and other polls found little attitudinal change.^10^

These studies assessed effects across the public overall. A significant proportion of the public was already very engaged with the issue, however: In the spring of 2015, 37% of Americans said they were “extremely” or “very sure” global warming is happening; close to a third (32%) said people in the United States are currently being harmed by global warming; and 11% said they were very worried.^11^ Audience segmentation identified 12% of the population as “alarmed”—people who are convinced of the reality and danger of climate change, fearful of its impacts, and are already taking action.^12^ Increased activism among these concerned groups could help motivate legislators to enact new environmental policies^13^ and companies to adopt more sustainable practices.^14^

This study focuses on the members of the public who are already highly concerned about climate change, to assess the influence of the Pope's climate leadership on their political and consumer activism. We first assess whether his leadership was a galvanizing force that increased consumer activism, political activism, and willingness to engage in political activism among this group, and then compare which forms of activism were most influenced, with the expectation that less difficult behaviors were more affected than those that are more difficult.

## 2 Exposure to the Pope's Message and Motivations for Collective Action

A large body of research shows that people are more likely to take action when they are *motivated*, and have the *ability* and *opportunity* to do so.^15^ We use the motivation‐ability‐opportunity (MOA) framework to identify some of the ways that exposure to the Pope's climate message could have influenced attitude‐behavior (A‐B) consistency (although we do not test the association of these predictors with consistency in this study) and to justify why examining the influence of the Pope's message on A‐B consistency is worth doing.

Hearing the Pope's climate change message is unlikely to have changed the audience's abilities or opportunities for activism.^16^ However, the Pope's message may have increased people's motivation to become activists by strengthening their beliefs and attitudes about global warming, connecting global warming to their personal values, message repetition, and prompting them to act.

### 2.1 Strengthening Beliefs and Attitudes

People are more motivated to act on their attitudes when attitudes are supported by beliefs that are readily accessible in memory at the time when a decision to act is made.^17^ Those who are convinced climate change is real, human‐caused, harmful, and solvable are more likely to engage in both political and consumer activism.^18^ Thus, the Pope's message, which emphasized the harmful impacts of climate change and the benefits of action, had the potential to increase the number and strength of beliefs that support climate‐friendly behavior—effectively bolstering the connection between people's attitudes toward the issue and their likelihood of taking political and consumer activism actions.

### 2.2 Connecting the Issue to Personal Values

The increased salience of personal values can also increase the motivation for people to act on their attitudes and beliefs. People may fail to act on their values if these values have been accepted unthinkingly from childhood as truisms—for example, the golden rule—but they may be motivated to do so when they consider how those values apply to a situation at hand.^19^ Social cognitive theory and the theory of moral disengagement also posit that intrinsic motivations such as moral or outcome expectancies (i.e., I will feel good about myself if I do X) are more powerful behavioral influences than extrinsic motivations like reducing risk and securing benefits.^20^

The Pope's message on climate change, which focused heavily on the immorality of the unequal impacts of climate change on the world's poor, introduced a new dimension of climate change to the public, connecting the issue to the widely held value that others should be treated equitably. By making the link between global warming and inequality, the Pope may have strengthened the personal relevance and motivational power of the issue, leading to an increased tendency to act.^19^

### 2.3 Repeating the Message

Additionally, the pope's repetition of these values and beliefs—over many months and through multiple channels—the encyclical, media appearances, public addresses, and even Twitter—may have made these beliefs and values more readily accessible in memory, which has been found to increase consistency between an individual's attitudes and behavior.^17^ His consistent outspokenness about the issue—including exhortations for political activism^21^ and ethical consumption^22^—may have broken through the wall of climate silence^23^ and increased perceptions that climate beliefs and action are normative,^24^ thereby increasing the motivation individuals may have had to translate their beliefs and attitudes into political and consumer actions.

### 2.4 Prompting Action

Finally, the pope may have increased motivation to act simply by asking people to take action.^25^ Invitations to act are powerful: for instance, interpersonal influence has been found to play an important role in activism, with many activists joining social movements in response to invitations from others.^26^ Prompts provide a focusing event which is a powerful occasion for attitudes to translate into behavior. Presumably, the stronger the pre‐existing attitudes, the more the prompts should elicit actions.

Given the multiple ways that the pope's message may have influenced people's motivations to act on their attitudes, we test the following research question:

*RQ1: Will greater exposure to the pope's message on climate change correlate with increased consistency between climate change attitudes and activism behaviors?*

We conceptualize attitude as a holistic orientation toward the issue of climate change comprised of a belief component (in this case, belief certainty that global warming is happening), an affective component (in this case, whether global warming is a good or bad thing), and a discrete emotion component (in this case, worry). This multidimensional conceptualization includes both affective and cognitive elements, in line with other conceptualizations of attitude that include both components.^27^

## 3 Collective Action and Behavior Difficulty

Our focus on political and consumer activism reflects two considerations: (a) these are among the most consequential actions people can take to promote mitigation^13^, ^14^ and (b) other proenvironmental behaviors—specifically, home energy use and transportation choices—are only weakly related to beliefs about global warming in the United States.^28^, ^29^ We discuss below the logic for this focus, and return to the MOA framework to discuss the reasons we expect stronger effects of exposure to the pope's message for some collective actions than others.

Agnone's analysis of environmental policy changes between 1960 and 1998 found that legislation is more likely to pass when public opinion is amplified by protests.^13^ Likewise, consumer boycotts that receive at least some national media attention have about a one‐in‐four success rate in influencing corporate practices; and for those that entail protests or demonstrations, the success rate jumps to about half.^14^ Ockwell et al.^30^ have argued that fostering public demand for government mitigation policies is the most effective way communication can be used to combat climate change—more effective than persuading people to conserve energy.^31^ The American public already favors action on climate change, with 66% of registered voters saying the United States should reduce its greenhouse gas emissions regardless of the actions of other nations, and 72% saying that corporations and industry should do more to address global warming in late 2016.^23^ Thus, amplification of the broad public's support for mitigation policy by activists may increase the likelihood of legislative and corporate change. Hence, collective action in the form of political and consumer activism may help motivate legislators and corporations to adopt more sustainable policies and practices by increasing the salience of public concern.

Of the two forms of activism, consumer activism is much more common in the United States. In the spring of 2015, 25% of Americans said that in the past year they had punished companies that oppose steps to mitigate climate change by not buying their products; by contrast, only 8% had contacted a legislator to voice support for mitigation.^27^ Among the alarmed segment of the population, these proportions are higher, but still well below what they could be: 59% had punished a company and only 26% had contacted a legislator.^27^

These differences in behavior frequency are likely to reflect both ability and opportunity. Ability encompasses both knowledge and skills.^17^ Most people have repeated, daily experience with shopping, about which they have acquired both. In contrast, relatively few Americans have been involved politically, beyond voting and signing petitions, so most lack knowledge of what to do and how to do it. Consumer activism also faces knowledge barriers, however: In 2008, 79% of the alarmed said they did not know which companies to target.^34^

Opportunity is likely to be higher for consumer activism than political activism as well, as virtually every household must purchase food and other household goods on a regular basis, with many opportunities to choose environment‐friendly products or services (as the pope noted). Political activism, by contrast, is not a part of normal everyday routines.

These baseline differences in behavior frequency are likely to affect the concerned public's behavioral responses to the pope. Behavior frequency has been used as a proxy indicator of the behavior's difficulty: easy behaviors are performed by large numbers of people while difficult behaviors are performed by few.^35^ Hence, given that consumer activism is three times as frequent in the United States as political activism, we can infer that consumer activism is less difficult. Kaiser et al. propose that the strength of the correlation between attitudes and behavior is linear, contingent on behavior difficulty, and increases monotonically such that the correlation is highest for the easiest to perform behaviors.^35^ The differences in behavior difficulty are therefore likely to translate into stronger correlations between climate change concern and consumer activism (which we operationalize as punishing or rewarding companies according to their behavior in regards to global warming), as compared to the correlation between concern and political activism (which we operationalize as contacting government officials and as willingness to join a campaign to persuade elected officials to enact global warming legislation). This difference is not likely to extend to respondents' expressed willingness to participate in collective action, however, as saying one is willing to join is far less difficult than actually doing so, and requires neither ability nor opportunity. Given these differences in the difficulty of activism behaviors, it is likely that the pope's message will be more apt to influence A‐B consistency with less difficult behaviors, relative to more difficult behaviors. In light of these considerations, we investigate the following research question:

*RQ2: Will the effects of exposure to the pope's climate change message on attitude‐behavior consistency be larger for less difficult behaviors?*

## 4 Analysis and Results

We investigated whether exposure to the Pope's message about global warming strengthened the relationship between four consumer and political activism behaviors by using within‐subject longitudinal data (where respondents were interviewed at two time points: spring 2015, Time 1 [T1] and fall 2015, Time 2 [T2]), which made it possible to isolate differences in behavior between the two time points, and to examine whether those differences were associated with exposure to the Pope's message. We predicted three behaviors (rewarding companies, punishing companies, contacting a government official) and one behavioral intention (willingness to join a campaign about climate change); predicting the behavior at T2 from the interaction of climate change attitude and exposure to the Pope's message—controlling for the individual's reported behavior at T1 (along with controls of age, education, income, gender, race, political ideology, and being Catholic or Christian). Thus, results show the differences in behavior at T2 by belief certainty and exposure to the Pope's message, controlling for the own person's likelihood of doing the behavior at T1. All three behaviors were measured dichotomously and were therefore modeled using logistic regression; the behavioral intention of willingness to join a campaign was modeled using linear regression. Hayes' PROCESS macro, model 1, in SPSS 24 was utilized to conduct the analysis.^36^

**Figure 1:**
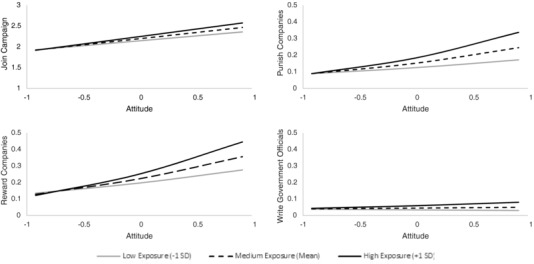
Relationship between attitude and four behaviors, at Time 2, as moderated by exposure to the Pope's message about global warming, controlling for likeliness of doing the behavior at Time 1. Note: The interactions between exposure to the Pope's message and attitude were significant for willingness to join a campaign, punishing companies, and rewarding companies, but not for writing government officials. The nonsignificant interaction is shown for reference. The figures for punishing companies, rewarding companies, and writing government officials show the conditional probabilities of performing those behaviors on the *y*‐axis across levels of attitude and exposure, while the figure for joining a campaign to convince elected officials to take action to reduce global warming is on the measured scale of that variable, ranging from 1 “I definitely would not do it” to 5 “I am participating in a campaign like this now.”

Three out of four models found evidence of an interaction between attitude and exposure to the Pope's message, such that those who had more exposure to the Pope's message had a stronger relationship between their climate change attitude and activism behaviors (RQ1; willingness to join a campaign, *b*_attitudeXexposure_ = 0.06, *p* < 0.05, rewarding companies, *b*_attitudeXexposure_ = 0.24, *p* < 0.05, and, at marginal significance, punishing companies *b*_attitudeXexposure_ = 0.25, *p* < 0.10, see **Table** **1** and **Figure** **1**). In other words, among those with stronger concern about global warming, more exposure to the Pope's message resulted in higher levels of the behavior at T2—even after controlling for T1 behavior—than among those who were less exposed. Specifically, when predicting willingness to join a campaign, attitude was related to behavior more strongly at high levels of exposure to the Pope's message (*b*_HighExposure_ = 0.356, *p* < 0.001) than at low levels of exposure to the Pope's views (*b*_LowExposure_ = 0.234, *p* < 0.001). Similarly, when predicting the likelihood of having punished a company due to their opposition to steps to address global warming, attitude was related to behavior more strongly at high levels of exposure to the Pope's message (*b*_HighExposure_ = 0.900, *p* < 0.001) than at low levels of exposure to the Pope's message (*b*_LowExposure_ = 0.407, *p* < 0.05); as it was also for predicting likelihood of having rewarded a company (*b*_HighExposure_ = 0.956, *p* < 0.001; *b*_LowExposure_ = 0.484, *p* < 0.05). The interaction predicting having contacted government officials (*b*_interaction_ = 0.25, *p* = 0.132) was not significant, however, the pattern of relationships for this outcome was similar to the rest of the outcomes. The overall pattern of results demonstrates that the political activism measure of contacting government officials—as a more difficult behavior—shows weaker effects than the consumer activism and political activism willingness measures (RQ2).

**Table 1:** Effect of global warming attitude on behavior, across levels of exposure to the Pope's message

	Interaction significant			Interaction not significant
	Join campaign	Punish companies	Reward companies	Contact government officials
Low exposure	0.234***	0.407*	0.484**	−0.125
Medium exposure	0.295***	0.652***	0.720***	0.113
High exposure	0.356***	0.900***	0.956***	0.352

*Note*: Entries represent the unstandardized coefficient of attitude predicting the behavior in that column at Time 2, conditioned on the individual's level of exposure to the Pope's message, and controlling for the individual's level of behavior at Time 1 (along with other controls). The significant interaction coefficients are: willingness to join a campaign, *b*_attitudeXexposure_ = 0.06, *p* < 0.05, rewarding companies, *b*_attitudeXexposure_ = 0.24, *p* < 0.05, and, at marginal significance, punishing companies *b*_attitudeXexposure_ = 0.25, *p* < 0.10. *p < .05, **p < .01, ***p < .001.

## 5 Conclusion

Overall, these findings suggest that hearing the Pope argue that climate action is a moral imperative led many people with prior high levels of concern to take additional activism actions—that is, their attitude‐behavior consistency increased (at lower levels of prior concern there was not an effect of the Pope's message). This is a meaningful effect of the Pope's message. While individual conservation actions are helpful, ultimately they are insufficient to adequately reduce carbon emissions. By contrast, activating collective political and consumer action will be essential to an effective global warming response.^30^

One caution is that all of our measures are self‐reported, and therefore potentially subject to the self‐report pitfalls of faulty recall and lack of precision. Additionally, as exposure to the Pope's message was measured, rather than manipulated, the results may be correlational, not causative—it may be that those who self‐reported higher exposure to the Pope's message were more likely to self‐report taking action, due to a tendency to simply self‐report higher across all survey measures. However, our research design offers some protection against this tendency; while some individuals may be likely to simply respond higher across all measures of a survey, there is no reason to suspect that likelihood would change over time. As our design uses within‐subject data, we control for an individual's prior levels of attitude and activism behavior—essentially partialing out an individual's tendency to respond highly across the survey, and increasing confidence that increased A‐B consistency can be linked to exposure to the Pope's message.

While this study does not examine the specific mechanisms by which the Pope's message strengthened attitude‐behavior consistency, his message may have increased people's motivation to become activists by strengthening their attitudes and beliefs about global warming, and connecting global warming more closely to their personal values. Higher exposure would have enhanced these effects, as the Pope repeated many times his central points: that climate change is harmful to the world's poor and that we are morally obligated to address it. The repetition of these points would have reinforced these views, making them more available in memory, while the Pope's promptings to consume more responsibly and pressurize governments to act would have directed his audience toward these specific responses. Testing these mechanisms as means by which leaders can motivate people to act on their attitudes is an important area for future research.

We also find that the relationship between attitudes and the least prevalent activism behavior we measured—contacting government officials—was unaffected by exposure to the Pope's message, in line with research that more difficult behaviors are less reflective of attitudes.^[27b,35]^ However, our measure of *willingness* to join a campaign to convince elected officials to take action did show the effect, which suggests that greater political participation could be stimulated if messages contain the information needed to take action. The literature suggests that motivation (or, in this case, willingness), must be paired with both *ability* and *opportunity* to result in action.^15^ As the results suggest that some individuals are motivated to act and are responsive to climate messaging, messages to highly concerned audiences should contain explicit information on how to participate and provide opportunities to do so. Recent social media campaigns in which protests, boycotts, and other collective actions are quickly organized are examples of this process in action.

Overall, these results suggest the potential for powerful new elite voices to inspire concerned individuals to take action, increasing their motivation to overcome the barriers that have previously inhibited them. Leadership and change management expert John Kotter has argued that: “Producing change is about 80% leadership—establishing direction, aligning, motivating, and inspiring people,” p. 14.^37^ This study finds that one such leader—Pope Francis—had the effect of inspiring those already convinced of climate change's seriousness to act on that belief. While the Pope's leadership is singular in its moral authority, visibility, and scale, leaders on a local level may have as great or even greater potential to inspire action: Local opinion leaders can model environmental leadership within a community, while articulating mutually held values. By drawing attention to the inequity of wealthy nations' actions harming the world's poor and highlighting this injustice, they may foster deeper thinking about climate impacts and solutions. Opinion leaders can provide a vision of a better future and help those around them overcome the social and cultural barriers to activism: As admired members of the community, they can be influential behavior models, teach others what actions to take, and draw more people into activist networks.^38^ Characteristics of climate leadership might include the articulation of mutual values and social and cultural norms that promote action^37^ and a vision of a better future—messages Pope Francis conveyed in *Laudato Sí* and his many speeches during his visit to the United States, which likely inspired many concerned Americans to take further climate action.

## 6 Experimental Section

*Sample*: Survey data for this study were collected from an online, nationally representative sample of American adults. The sample was drawn from GfK's KnowledgePanel, an online panel recruited using a combination of random digit dial and address‐based sampling techniques that cover virtually all (noninstitutional) resident phone numbers and addresses in the United States. Those contacted, who would choose to join the panel but do not have access to the Internet, are loaned computers and given Internet access so they may participate. The panel therefore includes a representative cross section of American adults—regardless of whether they have Internet access, use only a cell phone, etc.

The sample for this study was randomly selected from the panel, and received an invitation to participate, acceptance of which acknowledged informed consent. The first wave of data collection took place in February 27–March 10, 2015 (T1). All questionnaires were self‐administered by respondents in a web‐based environment, and approved by George Mason and Yale University human subjects review boards. The survey took, on average, about 26 min to complete, and asked a range of questions about respondents' climate change beliefs, attitudes, policy preferences, and religious and spiritual values. A total of 1263 panel members responded to the survey, for a completion rate of 57.7%.

In September, 2015, 1137 of the 1263 respondents remained active members of GfK's panel. All 1137 were recontacted between September 30 and October 19 for the second wave of data collection [T2]; 914 responded for a completion rate of 80.4%; of these, nine had completed the survey in less than 6 min and were dropped from the sample for a final *N* of 905.

Most of the questions on the recontact survey had been previously asked at T1. New questions were added concerning exposure and reactions to the Pope's encyclical on climate change and his visit to the United States. The survey was again conducted online and took an average of 22 min to complete. Respondents who dropped out between the first and second waves tended to be slightly younger, less educated, non‐Christians, non‐Republicans, and they tended to be more certain that global warming is happening. Prior to the analysis, missing data were hotdecked using Myers' hotdeck macro.^39^ Hotdecking assigns values to those missing by matching first on some characteristics (in this case sex and education) and then randomly selecting the value of an individual who matches on those characteristics. It has been shown to be more effective than listwise deletion.^40^

*Measures*: Climate change attitudes were measured with three items at T1: (1) a cognitive item read: “Do you think global warming is happening?”, with response options “yes,” “no,” and “don't know.” Those who responded yes or no were asked: “How sure are you that global warming is (not) happening?”, with response options “extremely sure,” “very sure,” “somewhat sure,” and “not at all sure.” Responses to these two items were collapsed into a nine‐point scale, ranging from 1 “extremely sure global warming is not happening” to 9 “extremely sure global warming is happening.” Those who responded “don't know” to the first question were assigned the midpoint of 5. The mean was 6.18, with a standard deviation of 2.39; (2) an affective item that read: “On a scale from −3 (very bad) to +3 (very good), do you think global warming is a bad thing or a good thing?” with response options ranging from “−3, very bad” (coded as 6) to “+3, very good” (coded as 1). The mean was 4.56, with a standard deviation of 1.31; and (3) a discrete emotion item that read: “How worried are you about global warming?”, with response options from “not at all worried”—coded 1—to “very worried—coded 4. The mean was 2.43, with a standard deviation of 0.93. A factor analysis of these three items was conducted (varimax rotation, maximum likelihood) and a single factor emerged (Cronbach's alpha = 0.75). The factor score was saved and utilized as the attitude variable (*M* = 0.00, SD = 0.92).

*Behavior*: Behavior was measured with four items measured at both T1 and T2. *Contacted government officials* was measured with a question that read: “Over the past 12 months, how many times have you done the following? [Written letters, e‐mailed, or phoned government officials about global warming]” and was coded 1 for contacting an official one or more times, and 0 for never or don't know (9.0% yes at T1 and 8.8% yes at T2). *Rewarded companies* was measured with a question that read: “Over the past 12 months, how many times have you done these things? [Rewarded companies that are taking steps to reduce global warming by buying their products]” and was coded 1 for rewarding companies one or more times, and 0 for never or don't know (27.7% yes at T1 and 28.8% yes at T2). *Punished companies* was measured with a question that read: “Over the past 12 months, how many times have you done these things? [Punished companies that are opposing steps to reduce global warming by NOT buying their products]” and was coded 1 for punishing companies one or more times, and 0 for never or don't know (19.6% yes at T1 and 21.9% yes at T2). *Willingness to join a campaign* was measured with a question that asked: “How willing or unwilling would you be to join a campaign to convince elected officials to take action to reduce global warming?” with response of “I definitely would not do it” (coded 1), “I probably would not do it” (coded 2), “I probably would do it” (coded 3), “I definitely would do it” (coded 4), and “I am participating in a campaign like this now” (coded 5). Those who responded “not sure” were coded 2.5, in between “I probably would not do it” and “I probably would do it.” The mean at T1 was 2.22 (SD = 0.88) and the mean at T2 was 2.22 (SD = 0.90).

*Exposure to the Pope's Views on Global Warming*: Exposure was measured with four items. A factor analysis of these four items was conducted (varimax rotation, maximum likelihood) and a single factor emerged (Cronbach's alpha = 0.85). The factor score was saved and utilized as the exposure variable (*M* = −0.02, SD = 0.98). Item wording is below.

The first item asked: “How much media coverage, if any, have you seen, read, or heard about Pope Francis' visit to the United States?”; the second and third asked, respectively: “How much media coverage, if any, about Pope Francis' views on the following have you seen, read, or heard in the past few months? [Global Warming; Protecting the Environment].” Answers for all three questions ranged from 1 “none/not sure/no answer” to 4 “a lot.” The fourth item of exposure to the Pope's message on global warming was an index of the various ways people could have encountered the Pope's climate message. All questions for this index were taken from the T2 data, with the exception of the first question reported below. The index was capped at 5 to reduce skew. All questions in this index, with the exception of question (c) had response options of “yes,” coded 1 and “no/not sure/no answer,” coded 0 (response options for question (c) are noted by the item). Questions included: (a) “This summer, Pope Francis is expected to release an encyclical (a letter sent to all Catholic Bishops worldwide), which will say that addressing global warming is a high priority for the Catholic Church. Before you read about it in this question, were you aware of this papal encyclical about global warming?”; (b) “This past summer, Pope Francis released an encyclical (a letter sent to all Catholic Bishops worldwide), which said that addressing global warming is a high priority for the Catholic Church. Before you read about it in this question, were you aware of this papal encyclical about global warming?”; (c) “How much, if at all, have the Pope's views on global warming been discussed in your place of worship?” [1 = A lot/some/a little; 0 = Not at all/does not apply to me/not sure/no answer]. Questions (d)–(j) began with the stem: “Have you heard the Pope speak about global warming or read direct quotes from him about global warming in any of the following ways?”; (d) “I have watched or listened to the Pope talking about global warming on television, radio, or the Internet”; (e) “I have read the Pope's words about global warming quoted in a nonreligious publication”; (f) “I have read the Pope's words about global warming quoted in a publication from my place of worship or another religious source”; (g) “I have heard the Pope's words about global warming quoted in my place of worship”; (h) “I have read excerpts or quotes from the Pope's encyclical about global warming”; (i) “I have read parts of the Pope's encyclical about global warming from the full document, either online, as a PDF, or in printed form”; and “(j) “I have read the Pope's encyclical about global warming in its entirety.”

*Controls*: Controls were measured at T1. *Age, education, income, gender*, and *race* (white or not) were provided by GfK. The average *age* was 51.8 (SD = 17.10). *Education* was measured on a 14‐point scale from “no formal education” (coded 1) to “professional or doctorate degree” (coded 14; *M* = 10.32, SD = 1.92). *Income* was measured a 19‐point scale from “less than $5000” (coded 1) to “$175 000 or more” (coded 19; *M* = 12.14, SD = 1.23). *Gender* was measured as male (50.5%, coded 0) or female (49.5%, coded 1). Race was coded as white (77.2%, coded 1) or not (22.8%, coded 0). *Political ideology* was measured with an item that asked: “In general, do you think of yourself as…” with response options ranging from “very liberal” (coded 1) to “very conservative” (coded 5), with a mean response of 3.19, closest to “moderate, middle of the road” (SD = 1.07). Religion was measured with the question: “What is your religion?”; and we included two dummy variables coding whether participants were *Catholic* (24.5%) or *Christian* (53.2%).

## Conflict of Interest

The authors declare no conflict of interest.
